# Effect of nursing students’ health literacy level on their knowledge and attitude toward epilepsy

**DOI:** 10.1590/1806-9282.20241808

**Published:** 2025-06-16

**Authors:** Kezban Koraş Sözen, Neziha Karabulut

**Affiliations:** 1 Niğde Omer Halisdemir University, Zubeyde Hanım Faculty of Health Sciences, Department of Surgical Nursing – Niğde, Turkey.; 2 Atatürk University, The Nursing Faculty, Department of Surgical Nursing – Erzurum, Turkey.

**Keywords:** Attitude, Epilepsy, Health literacy, Knowledge, Nursing

## Abstract

**OBJECTIVE:**

The aim of this study was to investigate the effect of nursing students’ health literacy level on their epilepsy knowledge and attitude.

**METHODS:**

Descriptive and cross-sectional study data were collected using a descriptive characteristics form, Epilepsy Knowledge Scale, Epilepsy Attitude Scale, and European Health Literacy Scale-Turkish adaptation.

**RESULTS:**

The mean Epilepsy Knowledge Scale and Epilepsy Attitude Scale scores were moderate, and the mean European Health Literacy Scale-Turkish adaptation score was adequate. The health literacy level significantly explained participants’ epilepsy knowledge.

**CONCLUSIONS:**

Epilepsy knowledge levels among nurses are intermediate, which reveals the fact that the nursing education curriculum should be revised. It is suggested that courses on health literacy impacting the level of knowledge and attitude toward epilepsy be added to the nursing education curriculum.

## INTRODUCTION

Epilepsy is a common primary brain disorder characterized by spontaneous, recurrent seizures that occur due to abnormal and excessive activities of cortical neurons^1^. Nurses who determine the needs of patients and provide care according to these needs should have sufficient knowledge and skills about epilepsy^2,3^.

Healthcare providers’ negative attitudes toward patients who have epilepsy may have negative effects on patients’ quality of life and their self-esteem. Therefore, nurses, who are among the most important healthcare providers, should have sufficient knowledge and skills about epilepsy^2,3^.

Health literacy is the knowledge, motivation, and competence to promote, maintain, and improve health; protect against diseases; and access, understand, evaluate, and apply information^4,5^. Nurses should strive to understand health ­literacy with their potential power to increase patients’ and families’ health literacy levels^6^.

The current study examined the effect of the level of health literacy among nursing students on their epilepsy knowledge (EK) and epilepsy attitude (EA).

## METHODS

### Study design and sample

The population of this descriptive and cross-sectional study consisted of 440 nursing students studying in the Zubeyde Hanım Faculty of Health Sciences at Niğde Omer Halisdemir University in the 2021–2022 academic year. It was aimed to reach the entire population without sample selection. The study was completed with 342 nursing students.

### Data collection

- Descriptive characteristics form: This form, prepared by the researcher in line with the literatüre^1,2^, included sociodemographic information of the participants.
- Epilepsy Knowledge Scale (EKS): This is a tool created by Aydemir in 2008, which was administered to 613 adults to assess the level of epilepsy awareness within the Niğde Omer Halisdemir population^7^. This scale is composed of 16 questions. Scores on the EKS can range from 0 to 16, with higher scores reflecting a greater understanding of epilepsy^7^.
- Epilepsy Attitude Scale (EAS): This was created by Aydemir in 2008, and is designed to assess the perceptions of Turkish society regarding epilepsy and those who have it^7^. This scale includes 14 items and the ­overall score can vary from 14 to 70. A higher score on the EAS suggests a more favorable attitude toward epilepsy and individuals living with epilepsy^7^.
- The European Health Literacy Scale-Turkish adaptation (EHLS-TR): This was developed by the European Health Literacy Research Consortium as part of the European Health Literacy Project from 2009 to 2012^8^. In 2016, Abacıgil et al. adapted this scale for Turkish populations. It has a scoring range from 47 to 188. To standardize the four indices for easier calculation and comparison, they are scaled to a metric from 0 to 50 using the following formula: Index=(mean-1)×(50/3) ^7,9^.

### Data analysis

The analysis of the data was conducted using the IBM SPSS Statistics 24.0 software, and descriptive statistics such as frequency, percentage, mean, and standard deviation were employed for the numerical data. The normal distribution of the data was assessed using the Shapiro-Wilk test. Analytical methods including Student's t-test, analysis of variance, Tukey's post-hoc test, Pearson's correlation, and simple linear regression analysis were utilized. A p-value of less than 0.05 was deemed to indicate statistical significance.

### Ethical considerations

The study was conducted after obtaining approval from the Ethics Committee of Niğde Omer Halisdemir University (meeting date: May 31, 2022, decision no. 2022/06-07), and institutional permission was granted from the institution where the study was conducted.

## RESULTS

Based on the distribution of the descriptive characteristics, the mean age of the participants was 21.03±2.02 years, 76% were male, 30.7% were 2nd-year students, 95.6% had ­previous knowledge about epilepsy, 87.4% did not have a family member with epilepsy, 56.4% had never seen an individual having an epileptic seizure, and 60.5% received first-aid training on epilepsy (Table 1). The mean EKS and EAS scores were ­moderate (9.11±3.85 and 54.35±7.65, respectively), and the mean EHLS score (37.31±6.61) was adequate.

**Table 1:** Comparison of students’ descriptive characteristics and scale scores.

Variables		n (%)	Mean scale scores (mean±SD)		
			EKS	EAS	EHLS
Gender					
	Female	260 (76)	9.48±3.53	55.98±6.69	37.74±6.77
	Male	82 (24)	7.59±4.22	53.17±7.63	35.87±5.89
	p		**0.000**	**0.003**	0.121
Grade					
	1st year	91 (26.6)	7.05±4.03	47.62±7.43	34.50±5.75
	2nd year	105 (30.7)	9.26±3.86	53.67±8.08	37.34±6.82
	3rd year	75 (21.9)	9.31±3.80	55.86±5.68	37.92±6.84
	4th year	71 (20.8)	9.90±3.43	54.13±8.62	38.42±6.04
	p		**0.000**	0.570	0.170
Have you heard or read information about epilepsy?					
	Yes	327 (95.6)	9.24±3.61	55.45±6.9	37.40±6.66
	No	15 (4.4)	4.47±4.75	52.27±9.41	33.42±0.18
	p		**0.002**	0.216	**0.000**
Do you have a family member with epilepsy?					
	Yes	43 (12.6)	10.60±3.15	55.14±7.08	36.98±8.15
	No	299 (87.4)	8.8±3.8	55.33±7.02	37.36±6.39
	p		**0.001**	0.867	0.808
Have you seen anyone having an epileptic seizure?					
	Yes	149 (43.6)	9.70±3.65	56.22±6.50	38.37±7.65
	No	193 (56.4)	8.50±3.83	54.59±7.34	36.53±5.66
	p		**0.003**	**0.030**	0.090
Have you received first-aid training or a course on epilepsy?					
	Yes	207 (60.5)	9.71±3.40	55.32±7.18	37.90±6.56
	No	135 (39.5)	7.97±4.11	55.28±6.80	36.25±6.60
	P		**0.000**	0.957	0.118

SD: Standard deviation; EKS: Epilepsy Knowledge Scale; EAS: Epilepsy Attitude Scale; EHLS: European Health Literacy Scale. Statistically significant values are denoted in bold.

As shown in Table 1, which shows the descriptive characteristics and scale scores of the students, female students had higher mean scores of EKS and EAS, and the difference was statistically significant. No significant difference was found between the mean scores of gender and the EAS. When the scale scores were compared according to education year, a statistically significant difference was found between the mean scores of the EKS and EAS. As a result of the Tukey honestly significant difference post-hoc test performed to find out from which group this difference originated, it was determined that the 1st-year students caused the difference. Having previous knowledge about epilepsy was found to cause a statistically significant difference in terms of the mean scores of the EKS and EHLS; however, no statistically significant difference was found in terms of the mean EAS score. Students who had a family member with epilepsy and who had seen someone ­having an epileptic seizure before were found to have higher EKS and EAS scores, and this difference was statistically significant; however, no statistically significant difference was found in terms of the mean EHLS score.

The mean scores of the students who received first-aid training on epilepsy were higher than those who did not, and the difference was statistically significant; however, the ­difference between the mean scores of EAS and EHLS was statistically insignificant.

There was a statistically significant and weak positive relationship between the mean total score of EKS and the mean total scores of the EAS and EHLS. No statistically significant relationship was found between the mean total score of EAS and the mean total score of the EHLS (Table 2).

**Table 2:** The relationship between the total mean scores of Epilepsy Knowledge Scale, Epilepsy Attitude Scale, and European Health Literacy Scale.

Relationship between the scales		EKS	EAS	EHLS
EKS	r	1	0.223**	0.242**
	p		**0.000**	**0.002**
EAS	r	0.223**	1	−0.021
	p	**0.000**		0.788
EHLS	r	0.242**	−0.021	1
	P	**0.002**	0.788	

** Correlation is significant at the 0.01 level (two-tailed).

EKS: Epilepsy Knowledge Scale; EAS: Epilepsy Attitude Scale; EHLS: European Health Literacy Scale. Statistically significant values are denoted in bold.

Based on Table 3, where the effect of the mean total score of EHLS on the EKS score was evaluated, the model was significant. Furthermore, the EHLS explained the EK level at a significant level, and an increase in EHLS score caused an increase in the EK level. When the effect of the EHLS total mean score on the EAS score was evaluated, it was found that the EHLS did not explain the level of EA significantly.

**Table 3:** Evaluation of the effect of the mean European Health Literacy Scale total score on the Epilepsy Knowledge Scale score using simple linear regression analysis.

	B	Std. error	β	t	P
Constant	3.858	1.656		2.329	0.021
EHLS	0.141	0.044	0.242	3.221	**0.002**

r=0.242; r^2^=0.058; F=10.376; p=0.002; Durbin-Watson statistic=1.643. EHLS: European Health Literacy Scale. Statistically significant values are denoted in bold. B, nonstandardized beta; β, standardized beta; t, significance of beta. R=0.242; R^2^=0.058; F=10.376; Durbin Watson static=1.643.

## DISCUSSION

The nursing students who participated in this study were found to have moderate levels of EK, EA, and adequate ­levels of epilepsy health literacy (EHL). Similar to the study performed by Unsar et al.^10^, this study found that the EK was at a medium level; however, the EK was at a low level in the study of Kiyak et al^11^. This may have resulted from regional educational differences. A review of the literature showed that the ET levels in previous studies were in parallel with those in this study^3,10,11^. While the level of EHL was in parallel with that in the study of Peksoy Kaya et al.^12^, the level of EHL was found to be insufficient in the study of Sukys et al^13^. Among the students participating in the present study, 95.6% stated that they had knowledge about epilepsy, 87.4% stated that their family members did not have epilepsy, 56.4% stated that they had never seen anyone ­having epileptic seizures, and 60.5% stated that they had received first-aid training on epilepsy. Similarly, a study by Shawahna et al.^2^ reported that nursing students had ­knowledge about epilepsy at low, medium, and high ­levels, 55.3% did not see anyone having an epileptic seizure, and 78.1% received first-aid ­training on epilepsy. The results of this study were consistent with the literature^10,11^.

In this study, female students had higher EK and EA levels than males; however, there was no difference between their EHL levels. The high levels of EC and EA in women can be attributed to their compassionate nature and social roles. Similarly, a study carried out by Unsar et al^10^. on nursing students found that female students had higher EK and EA levels than male students^10^. Contrary to these results, a study conducted by Yeni et al.^14^ on students receiving healthcare education reported no difference in EK and EA levels by gender. Similar to this study, Uysal et al.^15^ found no difference between EHL levels by gender, whereas Yılmaz et al.^16^ reported that female participants had a higher level of EHL.

In this study, those who had a family member with epilepsy had higher levels of EK than those who did not and those who received first-aid training on epilepsy had higher levels of EK than those who did not, and the difference was statistically significant. Similarly, the study conducted by Baltacı Göktaş et al.^17^ reported that the EK levels of those who received first-aid courses on epilepsy increased. This result is consistent with that in the literature^10,14,17^. There was no difference in the level of EHL between those who had a family member with epilepsy and those who did not, between those who received first-aid ­training on epilepsy and those who did not, and between students who had seen someone having an epileptic seizure before and those who had not. In general, while it was expected that the education received and experience would increase the level of EHL, a result contrary to the literature was found in this study. This may be thought to be due to the difference in the effectiveness of education and the effects of experience on individuals.

Students who had seen someone having an epileptic seizure before were found to have higher EK and EA levels; ­however, no significant difference was found in terms of the mean EHL score.

This study found that as the level of EK increased, the ­levels of EA and EHL increased, while the change in the level of EA did not affect the levels of EK and EHL. In this study, in which simple linear regression analysis was performed, individuals with a high level of EHL also had a high level of EK, and that EHL levels significantly explained EK levels. A study conducted by Yang^18^ reported that nurses with a high level of EHL were more prone to patient-centered care.

## CONCLUSION

Nursing students’ EK levels are intermediate, which reveals the fact that the nursing education curriculum should be revised. For this purpose, it is suggested that courses on health literacy impacting the level of knowledge be added to the nursing education curriculum, or new training programs should be organized.
